# Photon-efficient imaging with a single-photon camera

**DOI:** 10.1038/ncomms12046

**Published:** 2016-06-24

**Authors:** Dongeek Shin, Feihu Xu, Dheera Venkatraman, Rudi Lussana, Federica Villa, Franco Zappa, Vivek K. Goyal, Franco N. C. Wong, Jeffrey H. Shapiro

**Affiliations:** 1 Research Laboratory of Electronics, Massachusetts Institute of Technology, 77 Massachusetts Avenue, Cambridge, Massachusetts 02139, USA; 2 Dip. Elettronica, Informazione e Bioingegneria, Politecnico di Milano, Piazza Leonardo Da Vinci, 32, Milano I-20133, Italy; 3 Department of Electrical and Computer Engineering, Boston University, 1 Silber Way, Boston, Massachusetts 02215, USA

## Abstract

Reconstructing a scene's 3D structure and reflectivity accurately with an active imaging system operating in low-light-level conditions has wide-ranging applications, spanning biological imaging to remote sensing. Here we propose and experimentally demonstrate a depth and reflectivity imaging system with a single-photon camera that generates high-quality images from ∼1 detected signal photon per pixel. Previous achievements of similar photon efficiency have been with conventional raster-scanning data collection using single-pixel photon counters capable of ∼10-ps time tagging. In contrast, our camera's detector array requires highly parallelized time-to-digital conversions with photon time-tagging accuracy limited to ∼ns. Thus, we develop an array-specific algorithm that converts coarsely time-binned photon detections to highly accurate scene depth and reflectivity by exploiting both the transverse smoothness and longitudinal sparsity of natural scenes. By overcoming the coarse time resolution of the array, our framework uniquely achieves high photon efficiency in a relatively short acquisition time.

## Abstract (web summary)

Active optical imaging systems use their own light sources to recover scene information but typically operate with large number of photon detections. Here, the authors present a 3D imaging system that acquires depth and reflectivity information with a single photon camera operating in low-light conditions.

## Graphical abstract

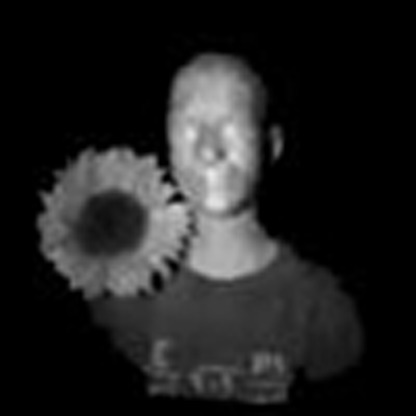

Active optical imaging systems use their own light sources to recover scene information. To suppress photon noise inherent in the optical detection process, they typically require a large number of photon detections. For example, a commercially available flash camera typically collects >10^9^ photons (10^3^ photons per pixel in a 1 megapixel image) to provide the user with a single photograph^1^. However, in remote sensing of a dynamic scene at a long standoff distance, as well as in microscope imaging of delicate biological samples, limitations on the optical flux and integration time preclude the collection of such a large number of photons^2^,^3^. A key challenge in such scenarios is to make use of a small number of photon detections to accurately recover the desired scene information. Exacerbating the difficulty is that, for any fixed total acquisition time, serial acquisition through raster scanning reduces the number of photon detections per pixel. Accurate recovery from a small number of photon detections has not previously been achieved in conjunction with parallel acquisition at a large number of pixels, as in a conventional digital camera.

Our interest is in the simultaneous reconstruction of scene three-dimensional (3D) structure and reflectivity using a small number of photons, something that is important in many real-world imaging scenarios^4^,^5^,^6^. Accurately measuring distance and estimating a scene's 3D structure can be done from time-of-flight data collected with a pulsed-source light detection and ranging system^7^,^8^,^9^. For applications specific to low-light-level 3D imaging, detectors that can resolve individual photon detections—from either photomultiplication^10^ or Geiger-mode avalanche operation^11^—must be used in conjunction with a time correlator. These time-correlated single-photon detectors provide extraordinary sensitivity in time-tagging photon detections, as shown by the authors of ref. 12, who used a time-correlated single-photon avalanche diode (SPAD) array to track periodic light pulses in flight.

The state-of-the-art in high photon-efficiency depth and reflectivity imaging was established by the authors of first-photon imaging (FPI)^13^, who demonstrated accurate 3D and reflectivity recovery from the first detected photon at each pixel. Their set-up, which used raster scanning and a time-correlated single SPAD detector, required exactly one photon detection at each pixel, making each pixel's acquisition time a random variable. Consequently, FPI is not applicable to operation using a SPAD camera^14^,^15^,^16^,^17^,^18^,^19^,^20^,^21^—all of whose pixels must have the same acquisition time—thus precluding FPI's reaping the marked image-acquisition speedup that the camera's detector array affords^12^,^22^,^23^. Although there have been extensions of FPI to the fixed acquisition-time operation needed for array detection^24^,^25^, both their theoretical modeling and experimental validations were still limited to raster scanning, with a single SPAD detector. As a result, they ignored the limitations of currently available SPAD cameras—much poorer time-tagging performance and pixel-to-pixel variations of SPAD properties—implying that these initial fixed acquisition-time (pseudo-array) frameworks will yield sub-optimal depth and reflectivity reconstructions when used with low-light experimental data from an actual SPAD camera.

Here we propose and demonstrate a photon-efficient 3D structure and reflectivity imaging technique that can deal with the aforementioned constraints that SPAD cameras impose. We give the first experimental demonstration of accurate time-correlated SPAD-camera imaging of natural scenes obtained from ∼1 detected signal photon per pixel on average. Unlike prior work, our framework achieves high photon efficiency by exploiting the scene's structural information in both the transverse and the longitudinal domains to censor extraneous (background light and dark count) detections from the SPAD array. Earlier works that exploit longitudinal sparsity only in a pixel-by-pixel manner require more detected signal photons to produce accurate estimates^26^,^27^. Because our new imager achieves highly photon-efficient imaging in a short data-acquisition time, it paves the way for dynamic and noise-tolerant active optical imaging applications in science and technology.

## Results

### Imaging set-up

**Figure 1:**
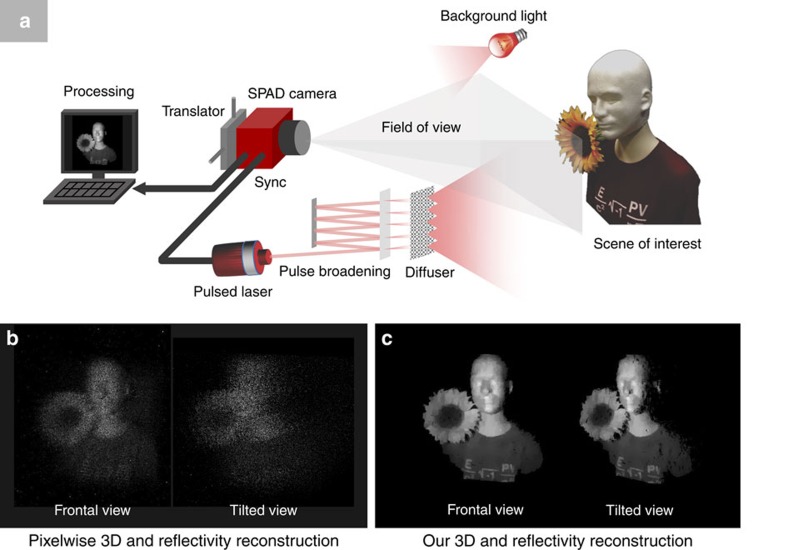
Single-photon array imaging framework. (**a**) SPAD-array imaging set-up. A repetitively pulsed laser flood illuminates the scene of interest. Laser light reflected from the scene plus background light is detected by a SPAD camera. Photon detections at each pixel are time tagged relative to the most recently transmitted pulse and recorded. The raw photon-detection data is processed on a standard laptop computer to recover the scene's 3D structure and reflectivity. (**b**) Example of 3D structure and reflectivity reconstruction using the baseline single-photon imager from ref. 22. (**c**) Example of 3D structure and reflectivity reconstruction from our processing. Large portions of the mannequin's shirt and facial features that were not visible in the baseline image are revealed using our method. Both images were generated using an average of ∼1 detected signal photon per pixel.

Our experimental set-up is illustrated in Fig. 1. The illumination source was a pulsed laser diode (PicoQuant LDH series with a 640-nm center wavelength), whose original output-pulse duration was increased to a full-width at half-maximum of ∼2.5 ns (that is, a root mean square (r.m.s.) value of *T*_p_≈1 ns). The laser diode was pulsed at a *T*_r_≈50 ns repetition period set by the SPAD array's trigger output. A diffuser plate spatially spread the laser pulses to flood illuminate the scene of interest. An incandescent lamp injected unwanted background light into the camera. The lamp's power was adjusted so that (averaged over the region that was imaged) each detected photon was equally likely to be due to signal (backreflected laser) light or background light. A standard Canon FL series photographic lens focused the signal plus background light on the SPAD array. Each photon detection from the array was time tagged relative to the time of the most recently transmitted laser pulse and recorded (Supplementary Methods).

The SPAD array^18^,^20^, covering a 4.8 × 4.8-mm footprint, consists of 32 × 32 pixels of fully independent Si SPADs and complementary metal-oxide-semiconductor (CMOS)-based electronic circuitry that includes a time-to-digital converter for each SPAD detector. The SPAD within each 150 × 150-μm pixel has a 30-μm-diameter circular active region, giving the array a 3.14% fill factor. At the 640-nm operating wavelength, each array element's photon-detection efficiency is ∼20% and its dark count rate is ∼100 Hz at room temperature. To extend the region that could be imaged and increase the number of pixels, we used multiple image scans to form a larger-size composite image. In particular, we mounted the SPAD array on a feedback-controlled, two-axis motorized translation stage to produce images with *N*_*x*_ × *N*_*y*_=384 × 384 pixels (Supplementary Figs 1a,b and 2a–c).

The SPAD array has a Δ=390 ps time resolution set by its internal clock rate. We set each acquisition frame length to 65 μs, with a gate-on time of 16 μs and a gate-off time of 49 μs for limiting power dissipation of the chip and for data transfer. At the start of each frame, the SPAD array was set to trigger the laser to generate pulses at a ∼20 MHz repetition rate. Hence, in the 16 μs gate-on time of each frame, ∼320 pulses illuminated the scene Supplementary Fig. 3).

### Observation model

We define 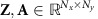 to be the scene's 3D structure and reflectivity that we aim to recover, and we let 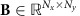 be the average rates of background-light plus dark-count detections. Flood illumination of the scene at time *t*=0 with a photon-flux pulse *s*(*t*) then results in the following Poisson-process rate function for (*i*,*j*)-th pixel of the composite image:


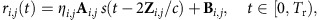


where *η*_*i*,*j*_∈(0,1] is the (*i*,*j*)th detector's photon-detection efficiency and *c* is the speed of light. Fabrication imperfections of the SPAD array cause some pixels to have inordinately high dark-count rates 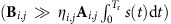, making their detection times uninformative in our imaging experiments because they are predominantly from dark counts. Thus we performed camera calibration to determine the set 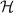 of these ‘hot pixels' (2% of all pixels in our case) so that their outputs could be ignored in the processing of the imaging data.

We define 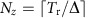 to be the total number of time bins in which the photon detections can be found, and let **C**_*i*,*j*,*k*_ be the observed number of photon counts in the *k*th time bin for pixel (*i*,*j*) after *n*_s_ pulsed-illumination trials. By the theory of photon counting^28^, we have that **C**_*i*,*j*,*k*_'s statistical distribution is


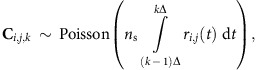


for *k*=1,2,…,*N*_*z*_, where we have assumed that the pulse repetition period is long enough to preclude pulse aliasing artifacts. Also, we operate in a low-flux condition such that ∑_*k*=1_^*N*^_*z*_**C**_*i*,*j*,*k*_, the total number of detections at a pixel, is much less than *n*_s_, the total number of illumination pulses to avoid pulse-pileup distortions. Our imaging problem is then to construct accurate estimates, 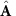 and 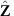, of the scene's reflectivity **A** and 3D structure **Z**, using the sparse photon-detection data 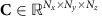.

### 3D structure and reflectivity reconstruction

In the low-flux regime, wherein there are very few detections and many of them are extraneous, an algorithm that relies solely on the aforementioned pixelwise photodetection statistics has very limited robustness. We aim to achieve high photon efficiency by combining those photodetection statistics with prior information about natural scenes.

Most natural and man-made scenes have strong spatial correlations among neighbouring pixels in both transverse and longitudinal measurements, punctuated by sharp boundaries^29^. While conventional works normally treat each pixel independently, our imaging framework exploits these correlations to censor/remove extraneous (and randomly distributed) photon-detection events due to background light and detector dark counts. It should be noted that unlike noise mitigation via spatial filtering and averaging, in which fine spatial features are washed out due to oversmoothing, our technique retains the spatial resolution set by the SPAD array. Our reconstruction algorithm optimizes between two constraints for a given set of censored measurements: that the 3D and reflectivity image estimates come from a scene that is correlated in both the transverse and longitudinal domains, and that the estimates employ the Poisson statistics of the raw single-photon measurements.

**Figure 2:**
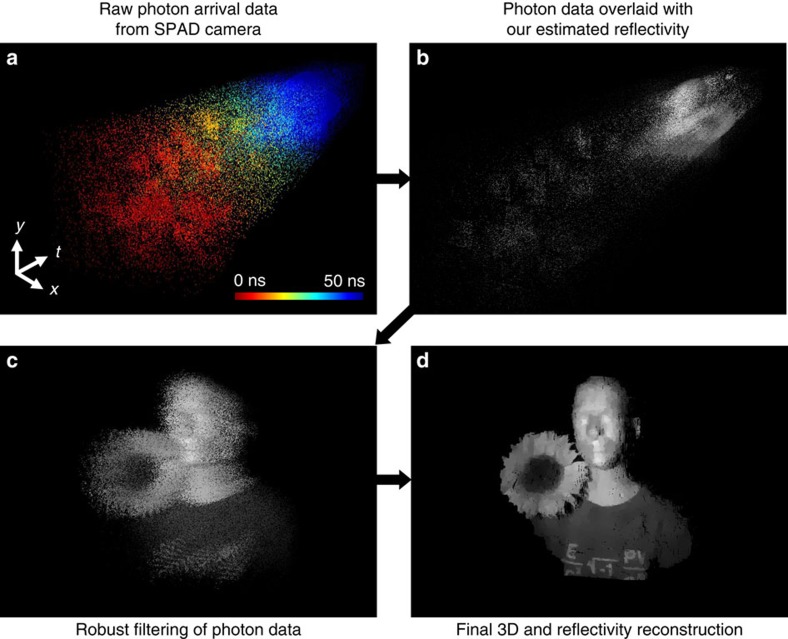
Stages of 3D structure and reflectivity reconstruction algorithm. (**a**) Raw time-tagged photon-detection data are captured using the SPAD camera set-up. Averaged over the scene, the number of detected signal photons per pixel was ∼1, as was the average number of background-light detections plus dark counts. (**b**) Step 1: raw time-tagged photon detections are used to accurately estimate the scene's reflectivity by solving a regularized optimization problem. (**c**) Step 2: to estimate 3D structure, extraneous (background light plus dark count) photon detections are first censored, based on the longitudinal sparsity constraint of natural scenes, by solving a sparse deconvolution problem. (**d**) Step 3: the uncensored (presumed to be signal) photon detections are used for 3D structure reconstruction by solving a regularized optimization problem.

The implementation of our reconstruction algorithm can be divided into the following three steps (Fig. 2; Supplementary Note 1).

Step 1: natural scenes have reflectivities that are spatially correlated—the reflectivity at a given pixel tends to be similar to the values at its nearest neighbours—with abrupt transitions at the boundaries between objects. We exploit these correlations by imposing a transverse-smoothness constraint using the total-variation (TV) norm^30^ on our reflectivity image. In this process, we ignore data from the hot-pixel set 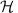. The final reflectivity image 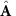 is thus obtained by solving a regularized optimization problem.

Step 2: natural scenes have a finite number of reflectors that are clustered in depth. It follows that in an acquisition without background-light or dark-count detections, the set of detection times collected over the entire scene would have a histogram with *N*_*z*_ bins that possesses non-zero entries in only a small number of small subintervals. This longitudinal sparsity constraint is enforced in our algorithm by solving a sparse deconvolution problem from the coarsely time-binned photon-detection data, which is specific to the array imaging set-up, to obtain a small number of representative scene depths. Raw photon-detection events at times corresponding to depths differing by more than *cT*_p_/2 from the representative scene depths are censored. As step 2 has identified coarse depth clusters of the scene objects, the next step of the algorithm uses the filtered set of photon detections to determine a high-resolution depth image within all identified clusters.

Step 3: similar to what was done in step 1 for reflectivity estimation, we impose a TV-norm spatial smoothness constraint on our depth image, where data from the hot-pixel set 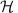 and censored detections at the remaining pixels are ignored. Thus, we obtain 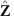 by solving a regularized optimization problem.

### Reconstruction results

**Figure 3:**
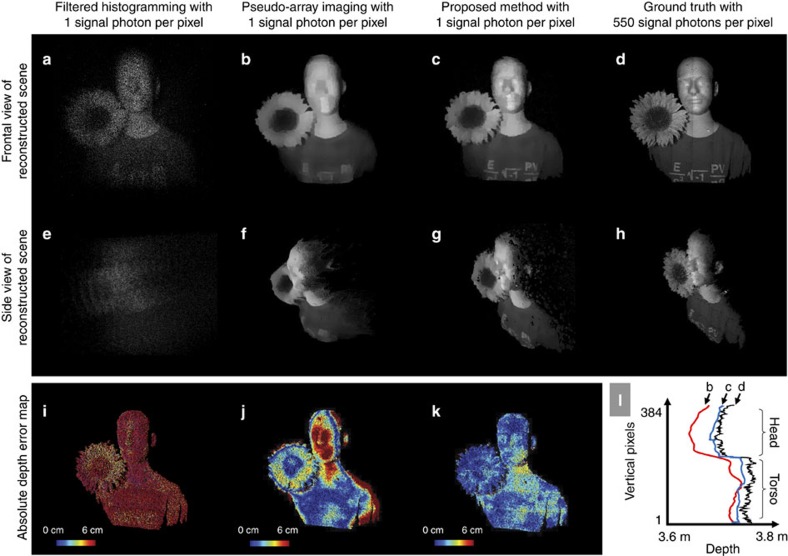
3D structure and reflectivity reconstructions. (**a**–**d**) Results of imaging 3D structure and reflectivity using the filtered histogram method, the state-of-the-art pseudo-array imaging method, our proposed framework and the ground-truth proxy obtained from detecting 550 signal photons per pixel. For visualization, the reflectivity estimates are overlaid on the reconstructed depth maps for each method. The frontal views, shown here, provide the best visualizations of the reflectivity estimates. (**e**–**h**) Results of imaging 3D structure and reflectivity from **a**–**d** rotated to reveal the side view, which makes the reconstructed depth clearly visible. The filtered histogram image is too noisy to show any useful depth features. The pseudo-array imaging method successfully recovers gross depth features, but in comparison with the ground-truth estimate in **h**, it overestimates the dimensions of the mannequin's face by several cm and oversmooths the facial features. Our SPAD-array-specific method in **g**, however, gives high-resolution depth and reflectivity reconstruction at low flux. (**i**–**k**) The depth error maps obtained by taking the absolute difference between estimated depth and ground-truth depth show that our method successfully recovers the scene structure with mean absolute error of 2 cm, which is sub-bin-duration resolution as *c*Δ/2≈6 cm, while existing methods fail to do so. (**l**) Vertical cross section plot of the middle of 3D reconstructions from pseudo-array imaging (red line), pixelwise ground truth (black line) and our proposed method (blue line). Note that our framework recovers fine facial features, such as the nose, while the pseudo-array imaging method oversmoothes them.

Figure 3 shows experimental results of 3D structure and reflectivity reconstructions for a scene comprised of a mannequin and sunflower when, averaged over the scene, there was ∼1 signal photon detected per pixel and ∼1 extraneous (background light plus dark count) detection per pixel. In our experiments, the per-pixel average photon-count rates of backreflected waveform and background-light plus dark-count response were 1,089 counts/s and 995 counts/s, respectively. The image resolution was 384 × 384 for this experiment. We compare our proposed method with the baseline pixelwise imaging method that uses filtered histograms^22^ and the state-of-the-art pseudo-array imaging method^25^.

From the visualization of reflectivity overlaid on depth, we observe that the baseline pixelwise imaging method (Fig. 3a,e) generates noisy depth and reflectivity images without useful scene features, owing to the combination of low-flux operation and high-background detections plus detector dark counts. In contrast, the existing pseudo-array method—which exploits transverse spatial correlations, but presumes constant *B*_*i*,*j*_—gives a reflectivity image that captures overall object features, but is oversmoothed to mitigate hot-pixel contributions (Fig. 3b). Furthermore, because the pseudo-array method presumes the 10-ps-class time tagging of a single-element SPAD that is used in raster-scanning set-ups, its depth image fails to reproduce the 3D structure of the mannequin's face from the ns-class time tagging afforded by our SPAD camera's detector array. In particular, it overestimates the head's dimensions and oversmooths the facial features (Fig. 3f), whereas our array-specific method accurately captures the scene's 3D structure and reflectivity (Fig. 3c,g). This accuracy can be seen by comparing our framework's result with the high-flux pixelwise depth and reflectivity images (Fig. 3d,h)—obtained by detecting 550 signal photons per pixel and performing time-gated pixelwise processing—that serve as ground-truth proxies for the scene's actual depth and reflectivity. For the fairest comparisons in Fig. 3, each algorithm—baseline pixelwise processing, pseudo-array processing and our new framework—had its parameters tuned to minimize the mean-squared degradation from the ground-truth proxies.

The depth error maps in Fig. 3i–k quantify the resolution improvements from our imager over the existing ones for this low-flux imaging experiment. Although the mean number of signal photon detections is ∼1 per pixel, in the high-reflectivity facial regions of the mannequin the average is ∼8 signal photon detections per pixel, while the number of signal photons detected at almost every pixel in the background portion of the scene is 0. Despite this fact, the pseudo-array imaging technique leads to a face estimate with high depth error due to oversmoothing incurred in its effort to mitigate background noise. It particularly suffers at reconstructing the depth boundaries with low-photon counts as well. Compared with conventional methods, our framework gives a much better estimate of the full 3D structure. We also study how the depth error of our framework depends on the number of photon counts at a given pixel. For example, our framework gives errors of 4.4 and 0.9 cm at pixels (260, 121) and (107, 187), which correspond to 1-photon-count depth boundary region and 8-photon-count mannequin face, respectively. Overall, we observe a negative correlation between the depth error and the number of photons detected at a pixel for our method (Supplementary Fig. 4). Recall that the time bin duration of each pixel of the SPAD camera is Δ=390 ps, corresponding to *c*Δ/2≈6-cm depth resolution. Overall, our imager successfully recovers depth with mean absolute error of 2 cm and thus with sub-bin-duration resolution, while existing methods fail to do so.

In terms of measuring the improvements in gray-scale imaging, we can compute the peak signal-to-noise ratio (PSNR) between the reference ground-truth reflectivity and the estimated reflectivity. While the PSNR values of the conventional pixelwise estimation and pseudo-array imaging are 19.3 and 24.9 dB, respectively, that of our method is 29.1 dB; it improves over both existing methods by at least 4 dB. We emphasize the difficulty of single-photon imaging in our set-up by computing that the SNR of the time-of-flight of a single photon ranges from −2.2 to 7.8 dB (Supplementary Note 2). We also note that varying the regularization parameters also affects the imaging performance (Supplementary Fig. 5). Lastly, the robustness of our reconstruction algorithm is evaluated by imaging an entirely different scene consisting of watering can and basketball, using regularization parameters pre-trained on the mannequin scene (Supplementary Fig. 6).

### Choice of laser pulse root mean square time duration

**Figure 4:**
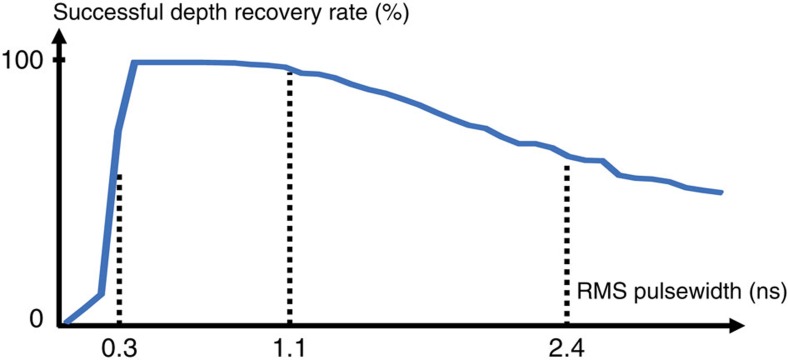
Relationship between r.m.s. pulse duration and depth-recovery accuracy. Plot of depth-recovery accuracy versus pulsewidth using our algorithm (steps 2 and 3) versus r.m.s. pulse duration *T*_p_ obtained by simulating a low-flux SPAD imaging environment with an average of 10 detected signal photons per pixel and 390-ps time bins. Depth recovery is deemed a success if estimated depth is within 3 cm of ground truth. In all our SPAD-array experiments, we used *T*_p_≈1 ns, which is in the sweet spot between durations that are too short or too long. We emphasize, however, that our algorithm is not tuned to a particular pulsewidth and can be performed using any *T*_p_ value.

For a transform-limited laser pulse, such as the Gaussian *s*(*t*) that our imaging framework presumes, the r.m.s. time duration *T*_p_ is a direct measure of system bandwidth. As such, it has an impact on the depth-imaging accuracy in low-flux operation. This impact is borne out by the simulation results in Fig. 4, where we see that the pulse waveform with the shortest r.m.s. duration does not provide the best depth recovery. Thus, in our experiments, we broadened the laser's output pulse to *T*_p_≈1 ns. The full-width at half-maximum is then 2.4 ns. This pulse duration allowed our framework to have a mean absolute depth error of 2 cm and resolve depth features well below the *c*Δ/2≈6 cm value set by the SPAD array's 390-ps-duration time bins (see Fig. 4 for details on depth-recovery accuracy versus r.m.s. pulse duration).

For application of our framework to different array technology, the optimal pulsewidth should scale with the SPAD camera's time-bin duration. For example, if our SPAD hardware were replaced to improve the timing resolution to the 50-ps range^17^, we would want to make sure the r.m.s. pulsewidth remains approximately three times longer than the time bin (or the full-width at half-maximum is approximately six times longer) based on our method for choosing optimal pulsewidth from Fig. 4. Thus, for accurate single-photon imaging with a 50-ps time-binning SPAD array, we would shorten our pulse from 1.1 ns to 140 ps.

## Discussion

**Figure 5:**
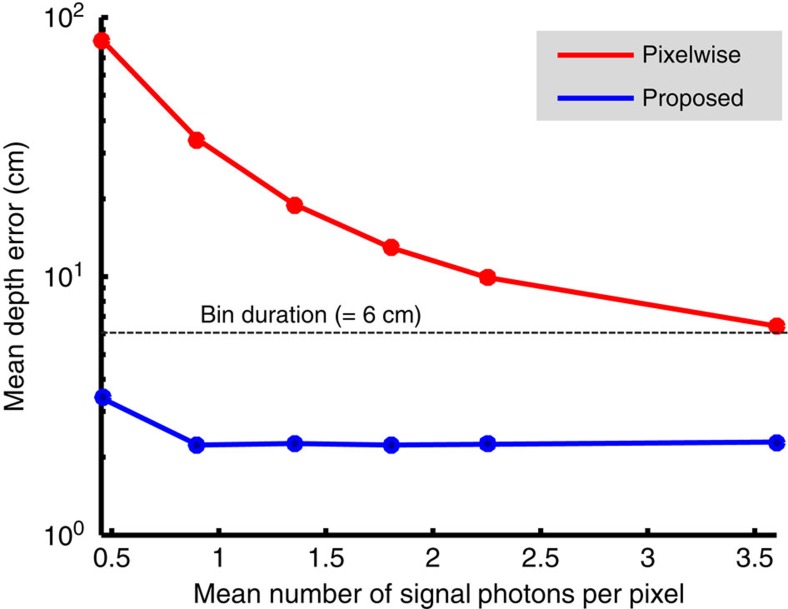
Photon efficiency of proposed framework. Plots of mean absolute depth error (log scale) versus average number of detected signal photons per pixel for imaging the mannequin and sunflower, using our proposed framework and the baseline pixelwise processor. Our method consistently realizes sub-SPAD-bin-duration performance throughout the low-flux region shown in the plot (for example, ∼2-cm error using 1 signal photon per pixel), whereas the baseline approach's accuracy is more than an order of magnitude worse, owing to its inability to cope with extraneous detections.

We have proposed and demonstrated a SPAD-camera imaging framework that generates highly accurate images of a scene's 3D structure and reflectivity from ∼1 detected signal photon per pixel, despite the presence of extraneous detections at roughly the same rate from background light and dark counts. By explicitly modeling the limited single-photon time-tagging resolution of SPAD-array imagers, our framework markedly improves reconstruction accuracy in this low-flux regime as compared with what is achieved with existing methods. The photon efficiency of our proposed framework is quantified in Fig. 5, where we have plotted the sub-bin-duration depth error it incurs in imaging the mannequin and sunflower scene versus the average number of detected signal photons per pixel. For this task, our algorithm realizes centimetre-class depth resolution down to <1 detected signal photon per pixel, while the baseline pixelwise imager's depth resolution is more than an order of magnitude worse because of its inability to cope with extraneous detections.

Because our framework employs a SPAD camera for highly photon-efficient imaging, it opens up new ways to image 3D structure and reflectivity on very short time scales, while requiring very few photon detections. Hence, it could find widespread use in applications that require fast and accurate imaging using extremely small amounts of light, such as remote terrestrial mapping^31^, seismic imaging^32^, fluorescence profiling^2^ and astronomy^33^. We emphasize, in this regard, that our framework affords automatic rejection of ambient-light and dark-count noise effects without requiring sophisticated time-gating hardware. It follows that our imager could also enable rapid and noise-tolerant 3D vision for self-navigating advanced robotic systems, such as unmanned aerial vehicles and exploration rovers^34^.

## Methods

### Reconstruction algorithm

Before initiating our three-step imaging algorithm, we first performed calibration measurements to: identify 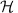, the SPAD array's set of hot pixels; obtain the average background-light plus dark-count rates for the remaining pixels; and determine the laser pulse's r.m.s. time duration (Supplementary Fig. 7a–c). For hot-pixel identification, we placed the SPAD camera in a dark room, and identified pixels with dark-count rate >150 counts/s, since the standard pixel of our camera should have a dark-count rate of 100 counts/s. The background-light plus dark-count rate at each pixel was identified by simply measuring the count rate, when the background-light source was on but the laser was off. Finally, the laser-pulse shape was calibrated by measuring the time histogram of 3,344 photon detections from a white calibration surface target that was placed ∼1 m away from the imaging set-up. It turned out that: ∼2% of our camera's 1,024 pixels were placed in 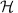; the background-light plus dark-count rates were indeed spatially varying across the remaining pixels; and the laser pulse's time duration was *T*_p_≈1 ns and reasonably approximated as a Gaussian.

We then proceed to step 1 of the reconstruction algorithm: we estimate reflectivity 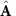 by combining the Poisson statistics of photon counts (Supplementary Fig. 8a) with a TV-norm smoothness constraint on the estimated reflectivity—while censoring the set of hot pixels—to write the optimization as a TV-regularized, Poisson image inpainting problem. This optimization problem is convex in the reflectivity image variable **A**, which allows us to solve it in a computationally efficient manner with simple projected gradient methods^35^. This step of the algorithm inputs a parameter *τ*_**A**_ that controls the degree of spatial smoothness of the final reflectivity image estimate. For a 384 × 384 image, the processing time of step 1 was ∼6 s on a standard laptop computer (Supplementary Note 1).

For step 2 of the reconstruction algorithm, we filtered the photon-detection data set to impose the longitudinal constraint that the scene has a sparse set of reflectors. This is because the scaled detection-time histogram hist(*c***T**/2) that has been corrected for the average background-light plus dark-count detections per bin is a proxy solution for hist(**Z**_Δ_), where hist(**Z**_Δ_) is a size–*N*_*z*_ histogram that bins the scene's 3D structure at the camera's *c*Δ/2 native range resolution. We used orthogonal matching pursuit^36^ on hist(*c***T**/2), the coarsely binned histogram of photon detections, to find the non-zero spikes representing the object depth clusters. This step of the algorithm requires an integer parameter *m* that controls the number of depth clusters to be estimated; here we used *m*=2, but our simulations show insensitivity to overestimation of the best choice of *m* (Supplementary Fig. 8b). We then discarded photon detections that implied depth values more than *cT*_p_/2 away from the estimated depth values, because they were presumably extraneous detections that are uniformly spread out during the acquisition time (Supplementary Fig. 8c). For a 384 × 384 image, the processing time of step 2 was ∼17 s on a standard laptop computer (Supplementary Note 1).

Having censored detections from all hot pixels and, through the longitudinal constraint, censored almost all extraneous detections on the remaining pixels, we treated all the uncensored photon detections as being from backreflected laser light, that is, that they were all signal photon detections. For step 3 of our reconstruction algorithm, we estimated the scene's 3D structure using these uncensored photon detections. Because we operated in the low-flux regime, many of the pixels had no photon detections and thus are non-informative for 3D structure estimation. A robust 3D estimation algorithm must inpaint these missing pixels, using information derived from nearby pixels' photon-detection times. Approximating the laser's pulse waveform *s*(*t*) by a Gaussian with r.m.s. duration *T*_p_, we solved a TV-regularized, Gaussian image inpainting problem to obtain our depth estimate 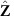. This is a convex optimization problem in the depth image variable **Z**, and projected gradient methods were used to generate 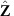 in a computationally efficient manner. This step of the algorithm inputs a parameter *τ*_**Z**_ that controls the degree of spatial smoothness of the final depth image estimate. For a 384 × 384 image, the processing time of step 3 was ∼20 s on a standard laptop computer (Supplementary Note 1).

### Code availability

The code used to generate the findings of this study is stored in the GitHub repository, github.com/photon-efficient-imaging/single-photon-camera.

### Data availability

The data and the code used to generate the findings of this study is stored in the GitHub repository, github.com/photon-efficient-imaging/single-photon-camera. All other Supplementary Data are available from the authors upon request.

## Additional information

**How to cite this article:** Shin, D. *et al*. Photon-efficient imaging with a single-photon camera. *Nat. Commun.* 7:12046 doi: 10.1038/ncomms12046 (2016).

## Supplementary Material

Supplementary Information Supplementary Figures 1-8, Supplementary Notes 1-2, Supplementary Methods and Supplementary References.

Supplementary Movie 1 This movie illustrates our proposed three-step algorithm. From noisy SPAD-camera data, we recover the depth and reflectivity of the mannequin-plus-sunflower scene in three steps: (1) estimate the scene reflectivity by solving a regularized optimization problem; (2) censor extraneous photon detections by solving a sparse deconvolution problem; (3) estimate the scene depth by solving a regularized optimization problem. The movie shows the reconstruction process described in Figure 2.

Supplementary Movie 2 This movie illustrates the photon efficiency of our proposed algorithm as compared to the conventional filtered-histogram method. With decreases in the number of detected photons, the proposed method substantially outperforms the conventional method in both reflectivity and depth reconstructions.

## References

[b1] Holst G. C. CCD Arrays, Cameras, and Displays JCD Publishing (1998).

[b2] Chen Y., Müller J. D., So P. T. & Gratton E. The photon counting histogram in fluorescence fluctuation spectroscopy. Biophys. J. 77, 553–567 (1999). 10388780 10.1016/S0006-3495(99)76912-2 PMC1300352

[b3] McCarthy A. . Long-range time-of-flight scanning sensor based on high-speed time-correlated single-photon counting. Appl. Optics 48, 6241–6251 (2009). 10.1364/AO.48.006241 19904323

[b4] Stettner R. in *SPIE Laser Radar Technology and Applications XV*, 768405 (Bellingham, WA, USA, 2010).

[b5] May S., Werner B., Surmann H. & Pervolz K. in *2006 IEEE/RSJ International Conference on Intelligent Robots and Systems*, 790–795 (Beijing, China, 2006).

[b6] Morris P. A., Aspden R. S., Bell J. E., Boyd R. W. & Padgett M. J. Imaging with a small number of photons. Nat. Commun. 6, 5913 (2015). 25557090 10.1038/ncomms6913 PMC4354036

[b7] Lee J., Kim Y., Lee K., Lee S. & Kim S.-W. Time-of-flight measurement with femtosecond light pulses. Nat. Photon. 4, 716–720 (2010).

[b8] Schwarz B. Lidar: Mapping the world in 3D. Nat. Photon. 4, 429–430 (2010).

[b9] Katz O., Small E. & Silberberg Y. Looking around corners and through thin turbid layers in real time with scattered incoherent light. Nat. Photon. 6, 549–553 (2012).

[b10] Buzhan P. . Silicon photomultiplier and its possible applications. Nucl. Instr. Meth. Phys. Res. Sect. A 504, 48–52 (2003).

[b11] Aull B. F. . Geiger-mode avalanche photodiodes for three-dimensional imaging. Lincoln Lab. J. 13, 335–349 (2002).

[b12] Gariepy G. . Single-photon sensitive light-in-flight imaging. Nat. Commun. 6, 6021 (2015). 25626147 10.1038/ncomms7021 PMC4338543

[b13] Kirmani A. . First-photon imaging. Science 343, 58–61 (2014). 24292628 10.1126/science.1246775

[b14] Becker W. Advanced Time-Correlated Single Photon Counting Techniques Springer (2005).

[b15] Richardson J. . in *2009 IEEE Custom Integrated Circuits Conference*, 77–80 (San Jose, California, USA, 2009).

[b16] Richardson J., Grant L. & Henderson R. K. Low dark count single-photon avalanche diode structure compatible with standard nanometer scale CMOS technology. IEEE Photonics Tech. Lett. 21, 1020–1022 (2009).

[b17] Veerappan C. . in *2011 IEEE International Solid-State Circuits Conference (ISSCC)*, 312–314 (San Jose, California, USA, 2011).

[b18] Villa F. . CMOS imager with 1024 SPADs and TDCs for single-photon timing and 3-D time-of-flight. IEEE J. Sel. Top. Quantum Electron. 20, 3804810 (2014).

[b19] Bronzi D. . 100,000 frames/s 64 × 32 single-photon detector array for 2D imaging and 3D ranging. IEEE J. Sel. Top. Quantum Electron. 20, 3804310 (2014).

[b20] Lussana R. . Enhanced single-photon time-of-flight 3D ranging. Opt. Express 23, 24962–24973 (2015). 26406696 10.1364/OE.23.024962

[b21] Bronzi D. . Automotive three-dimensional vision through a single-photon counting SPAD camera. IEEE Trans. Intell. Transp. Syst. 17, 782–795 (2016).

[b22] Buller G. S. & Wallace A. M. Ranging and three-dimensional imaging using time-correlated single-photon counting and point-by-point acquisition. IEEE J. Sel. Top. Quantum Electron. 13, 1006–1015 (2007).

[b23] Li D.-U. . Real-time fluorescence lifetime imaging system with a 32 × 32 0.13 *μ*m CMOS low dark-count single-photon avalanche diode array. Opt. Express 18, 10257–10269 (2010). 20588879 10.1364/OE.18.010257

[b24] Altmann Y., Ren X., McCarthy A., Buller G. S. & McLaughlin S. Lidar waveform based analysis of depth images constructed using sparse single-photon data. IEEE Trans. Image Process. 25, 1935–1946 (2016). 26886984 10.1109/TIP.2016.2526784

[b25] Shin D., Kirmani A., Goyal V. K. & Shapiro J. H. Photon-efficient computational 3D and reflectivity imaging with single-photon detectors. IEEE Trans. Comput. Imaging 1, 112–125 (2015).

[b26] Shin D., Shapiro J. H. & Goyal V. K. Single-photon depth imaging using a union-of-subspaces model. IEEE Signal Process. Lett. 22, 2254–2258 (2015).

[b27] Shin D., Xu F., Wong F. N. C., Shapiro J. H. & Goyal V. K. Computational multi-depth single-photon imaging. Opt. Express 24, 1873–1888 (2016). 26906766 10.1364/OE.24.001873

[b28] Snyder D. L. Random Point Processes Wiley (1975).

[b29] Besag J. On the statistical analysis of dirty pictures. J. Roy. Statist. Soc. Ser. B 48, 259–302 (1986).

[b30] Chambolle A., Caselles V., Cremers D., Novaga M. & Pock T. in *Theoretical Foundations and Numerical Methods for Sparse Recovery* (ed. Fornasier, M.) Ch. 9, 263–340 (Walter de Gruyter, 2010).

[b31] Côté J.-F., Widlowski J.-L., Fournier R. A. & Verstraete M. M. The structural and radiative consistency of three-dimensional tree reconstructions from terrestrial lidar. Remote Sensing Environ. 113, 1067–1081 (2009).

[b32] Haugerud R. A. . High-resolution lidar topography of the Puget Lowland, Washington. GSA Today 13, 4–10 (2003).

[b33] Gatley I., DePoy D. & Fowler A. Astronomical imaging with infrared array detectors. Science 242, 1264–1270 (1988). 17817072 10.1126/science.242.4883.1264

[b34] Watts A. C., Ambrosia V. G. & Hinkley E. A. Unmanned aircraft systems in remote sensing and scientific research: Classification and considerations of use. Remote Sens. 4, 1671–1692 (2012).

[b35] Harmany Z. T., Marcia R. F. & Willett R. M. This is SPIRAL-TAP: sparse Poisson intensity reconstruction algorithms—theory and practice. IEEE Trans. Image Process. 21, 1084–1096 (2012). 21926022 10.1109/TIP.2011.2168410

[b36] Pati Y. C., Rezaiifar R. & Krishnaprasad P. S. in *1993 Conference Record of The Twenty-Seventh Asilomar Conference on Signals, Systems and Computers*, Vol. 1, 40–44 (Pacific Grove, California, USA, 1993).

