# Supplementary material for: Photon-efficient imaging with a single-photon camera
Source: Nat Commun. 2016 Jun 24;7:12046. doi: 10.1038/ncomms12046 (PMC4931023; doi:10.1038/ncomms12046)
Supplement: Supplementary Information — Supplementary Figures 1-8, Supplementary Notes 1-2, Supplementary Methods and Supplementary References. [file ncomms12046-s1.pdf]

Supplementary Figures

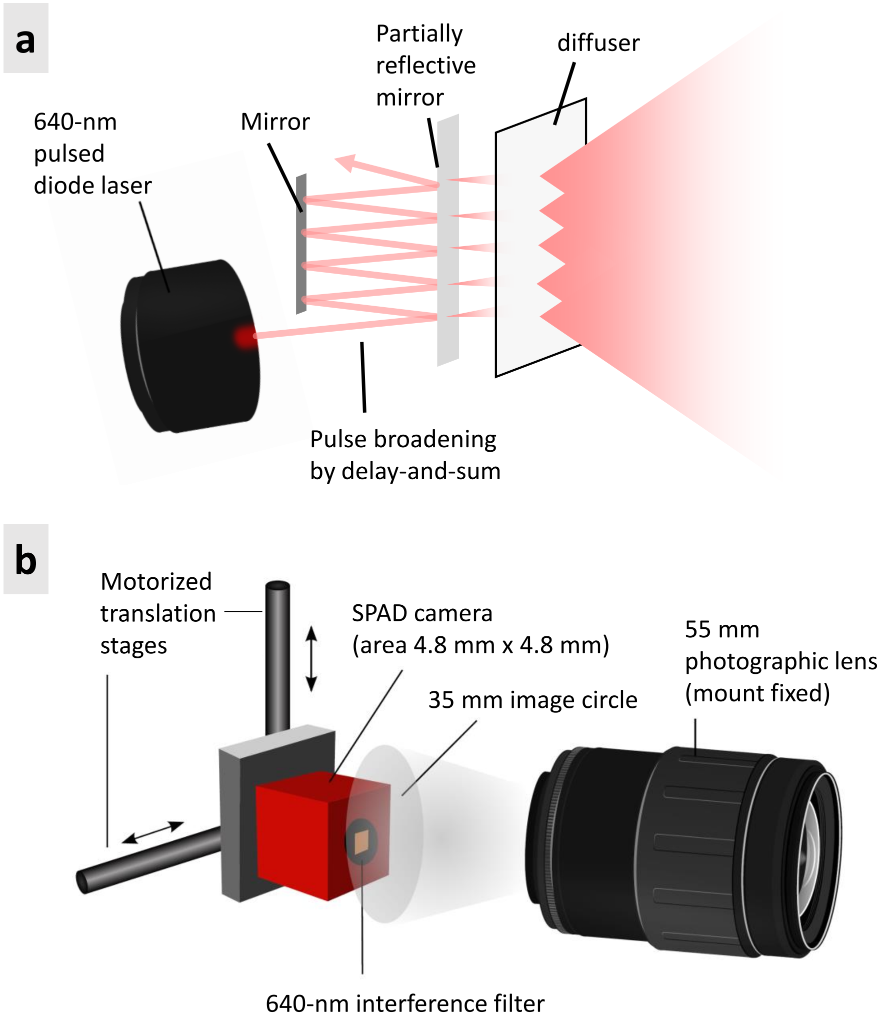

Supplementary Figure 1: Experimental SPAD-array imaging setup.

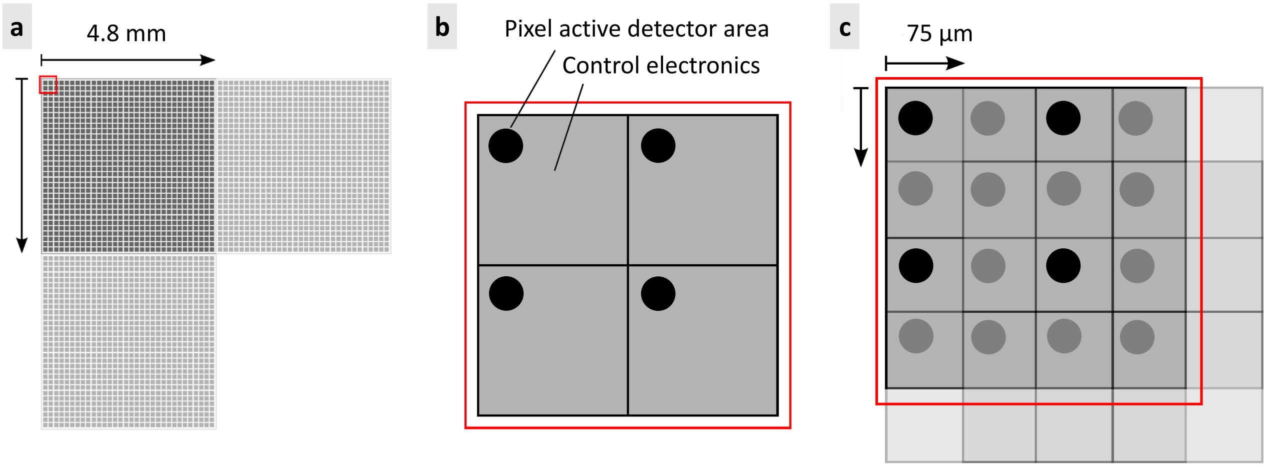

Supplementary Figure 2: Scanning scheme to obtain increased image area and image resolution using the 32×32-pixel SPAD array. (a) Array translation by increments of a full array size (4.8 mm) along both axes to image multiple tiles of the entire array. (b) Zoom-in view of individual SPAD pixels showing the active area with no sub-pixel scanning, (c) 2×2 sub-pixel scanning by translating along each axis by 75 μm, hence multiplying resolution by 2 in each dimension.

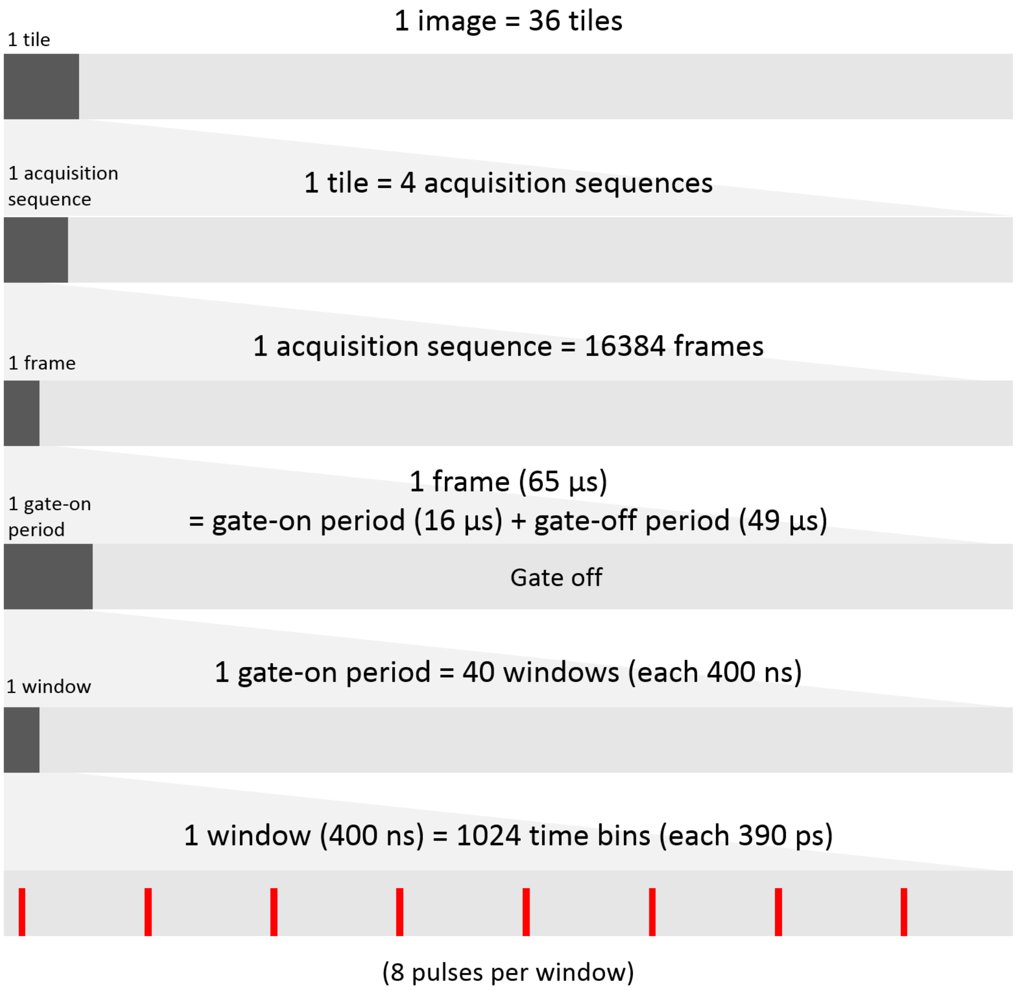

Supplementary Figure 3: SPAD-camera image acquisition terminology for readout-skip-mode operation.

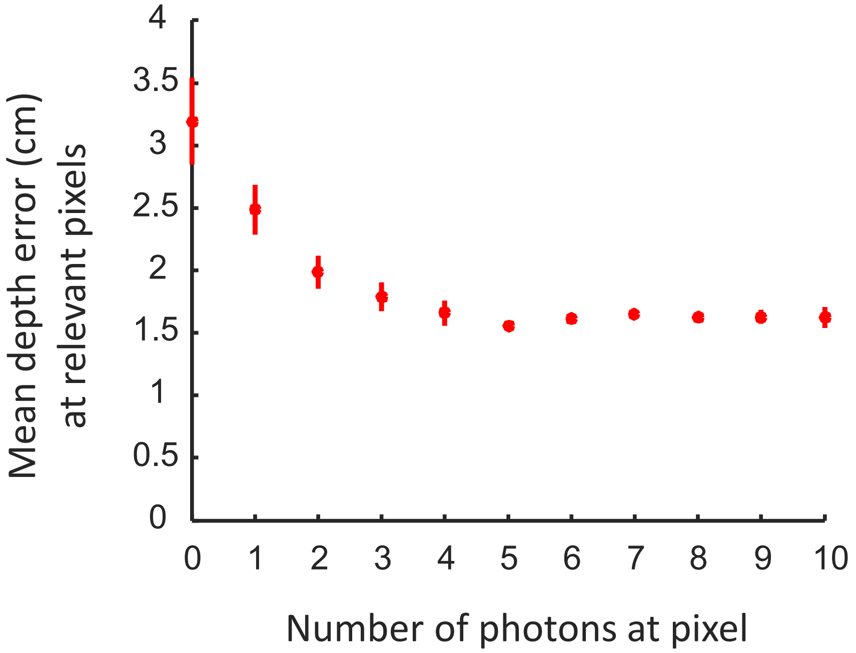

Supplementary Figure 4: Mean depth error of our framework vs. number of photons detected over all pixels of the mannequin and sunflower scene (bars indicate 95% confidence intervals). We observe for the high-photon-count regions, such as mannequin face, depth error can be low as 1.6 cm, while for the low-photon-count regions, such as object boundaries, the error is relatively higher. Note that because our framework is able to accurately impute depth values in pixels with zero photon detections using spatial regularization, it achieves finite depth error even for those no-detection pixels.

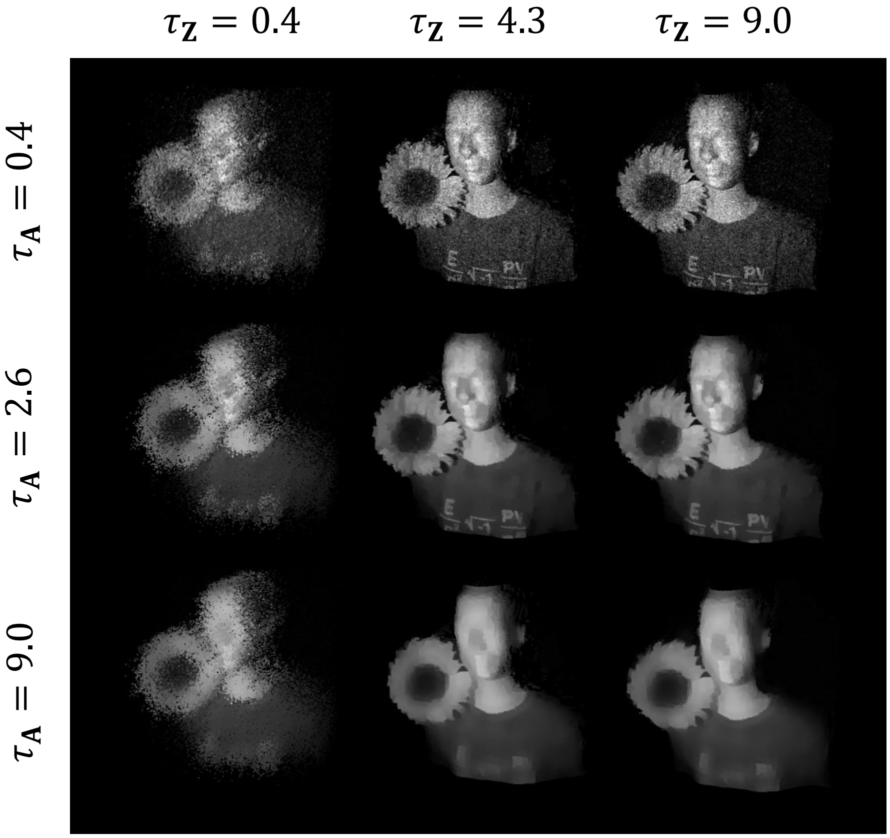

Supplementary Figure 5: Effect of varying the regularization parameters in our 3D structure and reflectivity reconstruction algorithm for the mannequin and sunflower scene. The optimal parameter pair was $\tau_{\mathbf{A}} = 2.6, \tau_{\mathbf{Z}} = 4.3$. We see that for small regularization parameters ($\tau_{\mathbf{A}} = 0.4, \tau_{\mathbf{Z}} = 0.4$), the reconstruction result is noisy due to undersmoothing. For high regularization parameters ($\tau_{\mathbf{A}} = 9.0, \tau_{\mathbf{Z}} = 9.0$), we find that the reflectivity estimate is almost featureless and the depth estimate has oversmoothing distortions that are visible at the sunflower's edges.

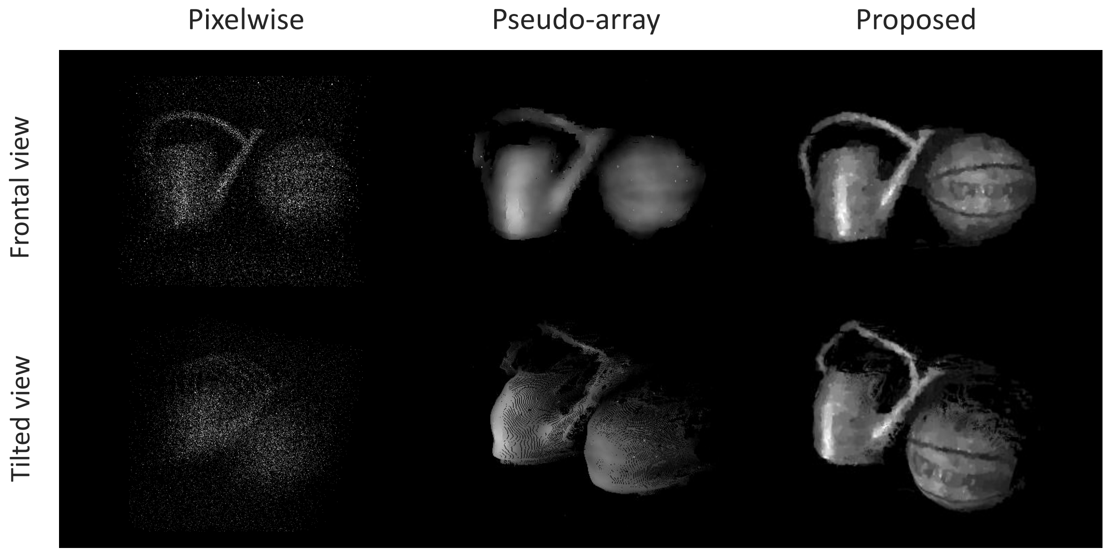

Supplementary Figure 6: Imaging results for the watering can and basketball scene. The training of the parameters of our algorithm was done offline using the mannequin and sunflower scene. Our 3D structure and reflectivity reconstruction method is compared against the baseline pixelwise imaging [1] and the pseudo-array method [2, 3]. The mean photon count averaged over all 384×384 pixels was approximately 1.0, and the mean signal photon count per pixel was approximately 0.5. Thus, the signal-to-background ratio was approximately 1, as in the mannequin and sunflower experiment. As the figure shows, our method successfully recovers the undistorted 3D shapes of the two objects and their reflectivity profiles, while the pixelwise method's image is exceedingly noisy and the pseudo-array's is oversmoothed in both depth and reflectivity.

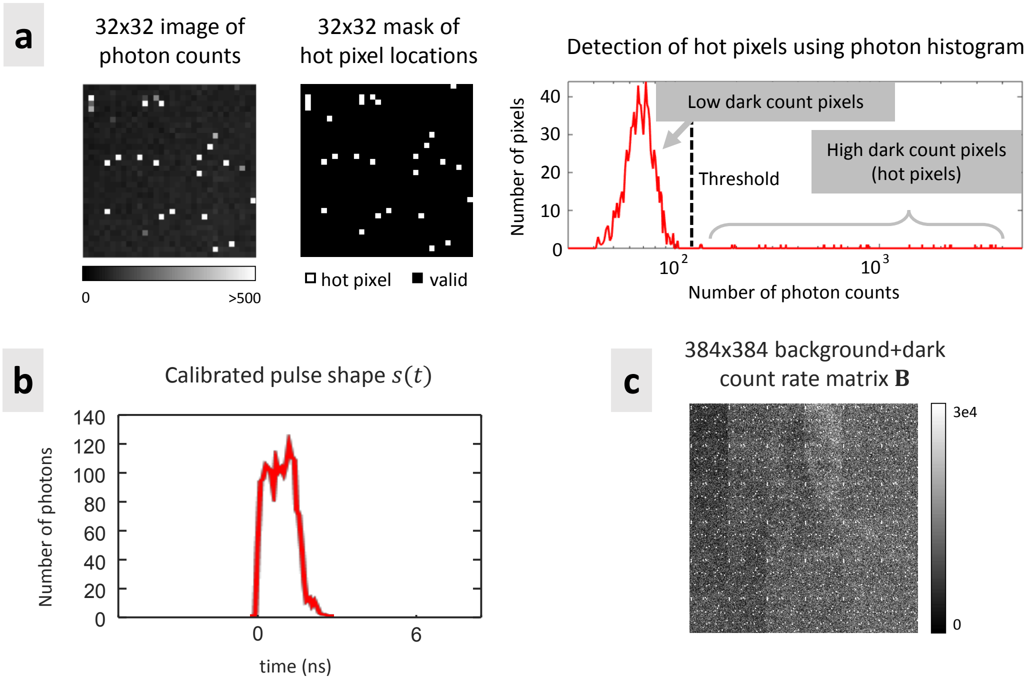

Supplementary Figure 7: Calibration results for the SPAD camera and scene parameters. (a) A 32×32 image of photon counts when the SPAD camera observes a weakly-reflecting planar wall with the laser off (left) was used to generate a 32×32 binary mask indicating hot-pixel locations with red markers (middle). The hot pixel mask was obtained by thresholding the photon-count image with an appropriate threshold chosen from the photon-count histogram (right). (b) Laser pulse shape. Extraneous photon detections were suppressed by time-gating near the roundtrip delay to a 1×1 m calibration target. (c) The non-constant 384×384 background-light plus dark-count rate matrix $\mathbf{B}$ for the mannequin and flower scene.

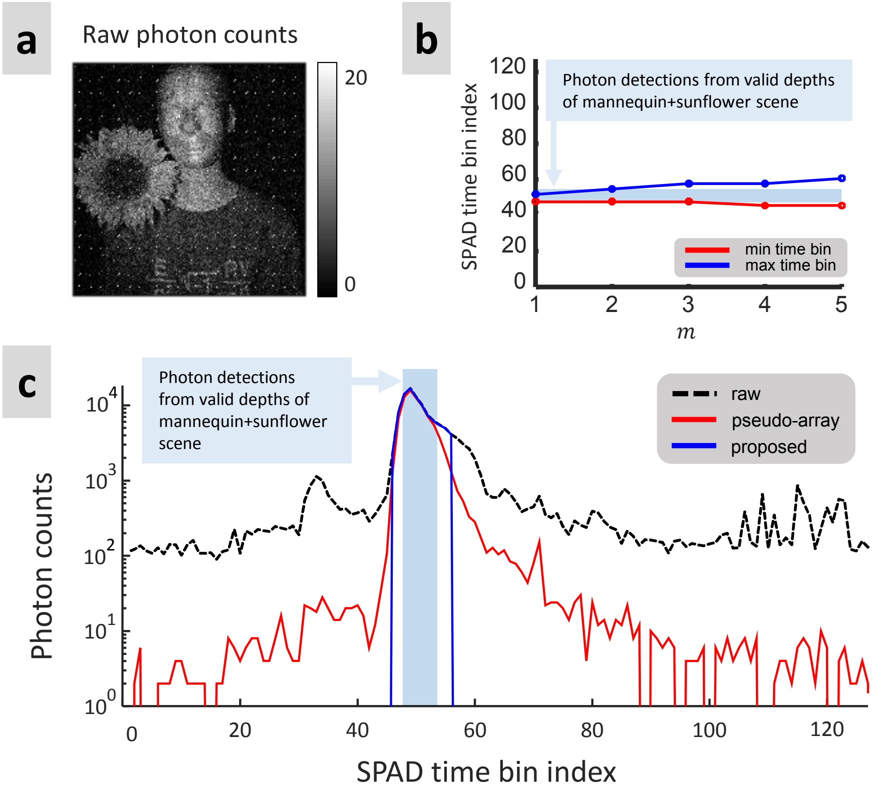

Supplementary Figure 8: (a) An image with each pixel indicating number of raw photon counts. (b) Estimated time interval for uncensored photons by Step 2 of reconstruction algorithm for various values of m. (c) Photon-count histograms after censoring extraneous detections for the state-of-the-art pseudo-array method [3] (red) and our method (blue) for the 384×384 mannequin and sunflower scene. The raw-data photon histogram is also given for reference (dashed black). By exploiting the scene's longitudinal sparsity, our method rejects more extraneous detections than does the pseudo-array method, which relies on transverse correlations. The greater the number of extraneous detections that survive censoring, the larger the best choice of regularization parameter in depth estimation, which will lead, in turn, to oversmoothing the depth image.

Supplementary Notes

Supplementary Note 1: Details of the reconstruction algorithm

Our reconstruction algorithm uses the sparse photon detection data cube $\mathbf{C} \in \mathbb{R}^{N_x \times N_y \times N_z}$ from the SPAD array to obtain image estimates of scene reflectivity $\mathbf{A} \in \mathbb{R}^{N_x \times N_y}$ and depth $\mathbf{Z} \in \mathbb{R}^{N_x \times N_y}$. The algorithm has three principal steps: estimating reflectivity, censoring extraneous detections, and estimating depth. Prior to the imaging experiment and processing, we perform preliminary measurements to characterize the set of hot pixels, the laser's pulse shape, and the background-light plus dark-count rate at each pixel. All computational data processing was performed using MATLAB in a laptop with an Intel(R) Core(TM) i7-4500u CPU running at 1.80 GHz.

Calibration

Hot pixel calibration

Hot pixels in the 32×32 pixel SPAD array are pixels whose dark-count rates are so high that their outputs are non-informative in estimating scene reflectivity and depth. To identify such pixels, we collected data with the laser turned off and the SPAD array observing a low-reflectivity target (dark wall). Under these conditions, hot pixels are clearly visible in the resulting photon-count image, see Supplementary Figure 7(a), and we get the SPAD array's hot-pixel set by a simple thresholding procedure on that image. With a threshold of 150 counts/s, 2% of the camera's 1024 pixels are labeled as hot pixels. (The dark count rate for a standard pixel was measured to be 100 counts/s during fabrication of the SPAD array chip.) The hot-pixel set $\mathcal{H}$ for the 384×384 pixel composite image is then obtained by appropriate translations of the array's hot pixels.

Laser pulse-shape calibration

To accurately measure the laser's pulse shape, we replaced our SPAD array—which has 390 ps time binning—with a single-pixel Micro Photon Devices SPAD detector and a HydraHarp time-correlator that provides 8 ps time binning. We focused the laser on a 1×1 m white planar calibration target placed $z \approx 1$ m from the imaging setup and obtained the photon-count histogram shown in Supplementary Figure 7(b) when 3344 signal photons were detected. Extraneous

photon detections were suppressed in this data collection by time-gating around $2z/c$ seconds. The laser's root mean-square (RMS) pulse duration was computed from this histogram to be $T_{\text{p}} \approx 1\,\text{ns}$. For our computational reconstruction algorithm, we use a Gaussian approximation to the calibrated pulse waveform, which is nearly symmetric and does not exhibit heavy tails. We chose to approximate the pulse waveform using a Gaussian mainly for computational efficiency reasons; because the Gaussian function has an expression that exponentiates a quadratic function, we can solve for our depth estimate by optimizing over the ℓ_2-loss function (the negative Gaussian log-likelihood), which is convex and has gradients that are easy to compute.

Background calibration

To calibrate the background-light plus dark-count rate matrix $\mathbf{B}$, we turned off the laser and obtained the $\mathbf{B}$ image, shown in Supplementary Figure 7(c), for the mannequin and flower scene. Spatial variations in $\mathbf{B}$ and the presence of hot pixels are clearly visible (note the periodic xy spacing of hot pixels, due to the translational scanning procedure).

We also studied the variation in background plus dark-count calibration measurements given that the laser is on. We measured that, with the laser on and off, we obtain an average of 0.473 and 0.453 extraneous photon counts per pixel, respectively. Because the difference between the two calibrated photon counts $(= 0.02)$ is much less than 1 photon, which is the smallest unit of our data that leads to varying scene reflectivity values, our proposed computational algorithm will be robust to the calibration being done with laser turned either on or off in our experimental setup.

Three-step image reconstruction algorithm

Step 1: Estimation of scene reflectivity

Using the statistics of the raw photon data cube $\mathbf{C}_{i,j,k}$, we have that $\mathbf{C}_{i,j}$, the total number of photon detections at pixel (i,j), is the sum of statistically independent Poisson random variables and hence it is itself Poisson, i.e.,

$$\mathbf{C}_{i,j} \equiv \sum_{k=1}^{N_z} \mathbf{C}_{i,j,k} \sim \text{Poisson}\left(\sum_{k=1}^{N_z} n_{\text{s}} \int_{(k-1)\Delta}^{k\Delta} r_{i,j}(t)\,dt\right) = \text{Poisson}\left(n_{\text{s}} \int_0^{T_{\text{r}}} r_{i,j}(t)\,dt\right). \tag{1}$$

The image of $\mathbf{C}$ is shown in Supplementary Figure 8(a). Using the expression given in the paper for $r_{i,j}(t)$ we then get

$$n_{\mathrm{s}} \int_0^{T_{\mathrm{r}}} r_{i,j}(t) = n_{\mathrm{s}}(\eta_{i,j} \mathbf{A}_{i,j} S + N_z \Delta \mathbf{B}_{i,j}), \tag{2}$$

where $S = \int_0^{T_{\mathrm{r}}} s(t - 2\mathbf{Z}_{i,j}/c)\, dt$. We can thus write $\mathbf{C}_{i,j}$'s probability mass function in terms of $\mathbf{A}_{i,j}$, as

$$\Pr(\mathbf{C}_{i,j}; \mathbf{A}_{i,j}) = \frac{[n_{\mathrm{s}}(\eta_{i,j} \mathbf{A}_{i,j} S + N_z \Delta \mathbf{B}_{i,j})]^{\mathbf{C}_{i,j}} \exp[-n_{\mathrm{s}}(\eta_{i,j} \mathbf{A}_{i,j} S + N_z \Delta \mathbf{B}_{i,j})]}{\mathbf{C}_{i,j}!}. \tag{3}$$

We let $\mathcal{L}_{\mathbf{A}}(\mathbf{A}_{i,j}; \mathbf{C}_{i,j}) = -\log \Pr(\mathbf{C}_{i,j}; \mathbf{A}_{i,j})$, where $\log$ denotes natural logarithm, be the negative log-likelihood of $\mathbf{A}_{i,j}$, and, for convenience, we set $S = 1$, because reflectivity images can be scaled arbitrarily. For robust reflectivity estimation, we first ignore data from the hot-pixel set $\mathcal{H}$ and then take our reflectivity image to be the non-negative $\hat{\mathbf{A}}$ that minimizes the negative log-likelihood of the remaining pixels plus a term that penalizes non-smoothness, i.e., we find $\hat{\mathbf{A}}$ by solving the following optimization problem:

$$\begin{aligned} \hat{\mathbf{A}} &= \arg\min_{\mathbf{A}} \left[\left(\sum_{(i,j) \notin \mathcal{H}} \mathcal{L}_{\mathbf{A}}(\mathbf{C}_{i,j}; \mathbf{A}_{i,j}) \right) + \tau_{\mathbf{A}} \left\| \mathbf{A} \right\|_{\mathrm{TV}} \right] \\ &\text{subject to} \qquad \mathbf{A}_{i,j} \geq 0, \qquad \text{for all } i, j. \end{aligned} \tag{4}$$

Here, $\tau_{\mathbf{A}}$ is the regularization parameter penalizing non-smoothness in the reflectivity image and $\| \cdot \|_{\mathrm{TV}}$ is the total-variation (TV) norm. The TV norm computes the sum of absolute gradients of the intermediate image solution; by minimizing the TV norm, we are searching for a reflectivity estimate that has small spatial variations overall and thus is spatially smooth as is true for natural scenes [4, 5]. The objective function in (4) is convex, so computing the reflectivity image can be done efficiently using projected-gradient methods [6].

We emphasize that our convex optimization formulation is not limited to using the total variation norm, but is flexible enough that the penalty term can be replaced with other scene priors that are suitable for particular low-light imaging applications. For example, when imaging a scene with a few point targets (e.g., biological imaging of extremely small cells occupying a few pixels), the ℓ_1-norm

may be used in the place of a total-variation norm in our optimization framework to exploit the transverse-sparse distribution of scene targets instead of their spatial smoothness [7, 8].

We chose the regularization parameter by first generating reflectivity images with $\tau_{\mathbf{A}} = \beta_{\mathbf{A}}/(1-\beta_{\mathbf{A}})$ for each $\beta_{\mathbf{A}} \in \{0.1, 0.2, 0.3, \ldots, 0.9\}$ and then choosing the $\tau_{\mathbf{A}}$ value that minimized ℓ_2 distance from the ground truth reflectivity. When ground truth is not available, as would be true in practical imaging scenarios, the regularization parameter can be chosen using cross-validation methods [9, 10, 11].

Step 2: Censoring extraneous detections

Unlike reflectivity estimation, which in penalized maximum-likelihood form yields an optimization problem that is convex even in the presence of extraneous detections from background light and dark counts, the optimization problem for penalized maximum-likelihood depth estimation when there are extraneous detections is not convex, and hence not computationally tractable [3]. Thus, in order to obtain a tractable problem for depth estimation, we first censor extraneous detections. By assuming that the uncensored detections are indeed due to the active illumination backreflected from the scene, we obtain a convex optimization problem.

Let $\mathbf{z}_\Delta = \mathrm{hist}(\mathbf{Z}_\Delta)$ be the one-dimensional histogram of the scene's unknown depth values, $\mathbf{Z}$, in N_z bins of width $c\Delta/2\,\mathrm{m}$ determined by the detector's time-tagging resolution. A natural assumption is that $\mathbf{z}_\Delta$ is sparse, because opaque and reflective objects are individually clustered in depth. Thus, by locating the non-zero elements of $\mathbf{z}_\Delta$ we can be very effective in censoring the non-informative extraneous detections, because they are uniformly distributed over $[0, T_{\mathrm{r}})$.

We obtain a proxy estimate for $\mathbf{z}_\Delta$ using our raw photon data cube as follows. We compute $\tilde{\mathbf{z}}_\Delta = \mathrm{hist}(c\mathbf{T}/2)$, where $\mathbf{T}$ is the set of raw detection times obtained by ignoring hot pixels, summing $\mathbf{C}_{i,j,k}$ over its transverse-coordinate indices, and then subtracting out the average background-light plus dark-count response, viz.,

$$(\tilde{\mathbf{z}}_\Delta)_k = \sum_{(i,j)\notin\mathcal{H}} (\mathbf{C}_{i,j,k} - \Delta\mathbf{B}_{i,j}), \qquad k = 1, \ldots, N_z. \tag{5}$$

We note that $\tilde{\mathbf{z}}_\Delta$ is a noisy version of $\mathbf{z}_\Delta$ convolved with $s_z \propto s(2z/c)$, where $s(t)$ is the laser's pulse shape and $\int s_z\,dz = 1$. Thus, the optimization problem that

solves for the sparse signal $\hat{\mathbf{z}}_\Delta$ from raw detection counts $\mathbf{C}$ is

$$\begin{aligned} \hat{\mathbf{z}}_\Delta \; &= \arg\min_{\mathbf{z}_\Delta} \quad \sum_{k=1}^{N_z} \|(\tilde{\mathbf{z}}_\Delta)_k - (\mathbf{s}_z \star \mathbf{z}_\Delta)_k\|_2^2 \qquad (6) \\ &\text{subject to} \quad \|\mathbf{z}_\Delta\|_0 = m, \quad (\mathbf{z}_\Delta)_k \geq 0, \quad \text{for all } k = 1, \ldots, N_z, \end{aligned}$$

where $\mathbf{s}_z$ is s_z discretized into bins of width $c\Delta/2$, $\star$ is the discrete convolution operator, $\|\mathbf{z}_\Delta\|_0$ is the ℓ_0-norm of $\mathbf{z}_\Delta$, and m is the number of non-zero elements in the reconstructed $\hat{\mathbf{z}}_\Delta$, which represents the centers of our depth clusters of interest. In our experiments, we chose $m = 2$ because our scene of interest consisted of a mannequin and a flower with a small separation in depth. We note that overestimating the number of depth clusters m does not significantly degrade the final depth reconstruction result heavily, as multiple neighboring depth clusters can be used to reject as many extraneous counts as a single depth cluster. It can also resolve the issue of an object's occupying a large longitudinal space, such that a single depth cluster is not sufficient to characterize it. Supplementary Figure 8(b) shows that even when m is overestimated, the estimated set of time intervals of uncensored photons does not increase very much, and only a small number of extraneous counts is left uncensored.

On the other hand, when the number of depth clusters is underestimated compared to the true number of depth clusters (e.g., Step 2 looking for one cluster given a scene with two objects separated in depth), then the algorithm will be biased towards recovering the 3D structure of the brighter object.

The optimization program in (6) is a discrete sparse-deconvolution problem that we solve approximately using a modified version of orthogonal matching pursuit (OMP) [12]. OMP is a fast and robust sparse signal-pursuit algorithm, which we modified to orthogonally project the intermediate solution onto the nonnegativity constraint set at every iteration.

Let $\text{supp}(\hat{\mathbf{z}}_\Delta) = \{k : (\hat{\mathbf{z}}_\Delta)_k \neq 0, k = 1, 2, \ldots, N_z\}$ be the support of the solution to (6). Because signal-photon detections at pixel (i, j) are mostly confined to T_p seconds around $2Z_{i,j}/c$, we use

$$\mathcal{S}_{T_\text{p}} = \{k : |k - k'| < T_\text{p}/\Delta, \text{ for any } k' \in \text{supp}(\hat{\mathbf{z}}_\Delta)\}. \qquad (7)$$

to generate the photon-sparse data cube after censoring, denoted $\tilde{\mathbf{C}} \in \mathbb{R}^{N_x \times N_y \times N_z}$, via

$$\tilde{\mathbf{C}}_{i,j,k} = \begin{cases} \mathbf{C}_{i,j,k}, & \text{if } k \in \mathcal{S}_{T_\text{p}} \text{ and } (i, j) \notin \mathcal{H}, \\ 0, & \text{otherwise.} \end{cases} \qquad (8)$$

Our extraneous-detection censoring mechanism differs significantly from the ones used in first-photon imaging [13] and pseudo-array imaging [2, 3]. Those prior works exploited transverse correlations in a scene to discriminate extraneous detections from signal detections, whereas our method relies on the longitudinal sparsity of natural scenes when they are subjected to the coarse time-binning inherent in a SPAD array. Supplementary Figure 8(c) shows the increased censoring of extraneous detections—for the mannequin and flower dataset—that results from using our method instead of the pseudo-array method.

Step 3: Estimation of scene depth

Assuming that $\tilde{\mathbf{C}}_{i,j,k}$ contains only the signal photon detections, the detection time-bin $T_{i,j}$ at pixel $(i,j) \notin \mathcal{H}$ has probability mass function

$$\Pr(T_{i,j};\mathbf{Z}_{i,j}) = \frac{\displaystyle\int_{(T_{i,j}-1)\Delta}^{T_{i,j}\Delta} s(t-2\mathbf{Z}_{i,j}/c)\,dt}{\displaystyle\sum_{T'_{i,j}=1}^{N_z}\int_{(T'_{i,j}-1)\Delta}^{T'_{i,j}\Delta} s(t-2\mathbf{Z}_{i,j}/c)\,dt}, \qquad T_{i,j} = 1,2,\ldots,N_z. \quad (9)$$

Approximating the laser's pulse shape $s(t)$ by a Gaussian, and using left-Riemann sums to approximate the integrals, we can reduce the negative log-likelihood function $\mathcal{L}_{\mathbf{Z}}(\mathbf{Z}_{i,j};T_{i,j}) = -\log\Pr(T_{i,j};\mathbf{Z}_{i,j})$ to the simple ℓ_2-loss function:

$$\mathcal{L}_{\mathbf{Z}}(\mathbf{Z}_{i,j};T_{i,j}) = \begin{cases} \|T_{i,j}\Delta - 2\mathbf{Z}_{i,j}/c\|_2^2, & (i,j)\notin\mathcal{H}, \\ 0, & \text{otherwise.} \end{cases} \quad (10)$$

For robust depth estimation, we take our depth image to be the non-negative $\hat{\mathbf{Z}}$ that minimizes the negative log-likelihood plus a term that penalizes non-smoothness, viz., we find $\hat{\mathbf{Z}}$ by solving the following optimization problem:

$$\hat{\mathbf{Z}} = \arg\min_{\mathbf{Z}} \left[\sum_{(i,j)\notin\mathcal{H}}\sum_{T_{i,j}\in\mathbf{U}_{i,j}} \mathcal{L}_{\mathbf{Z}}(\mathbf{Z}_{i,j},T_{i,j})\right] + \tau_{\mathbf{Z}}\,\|\mathbf{Z}\|_{\text{TV}} \quad (11)$$
$$\text{subject to} \qquad \mathbf{Z}_{i,j} \geq 0, \qquad \text{for all } i,j,$$

where $\mathbf{U}_{i,j}$ is the set of uncensored detection times at pixel (i,j) and $\tau_{\mathbf{Z}}$ is a regularization parameter penalizing non-smoothness in the depth image. The problem in (11) is convex, so it can be solved efficiently using projected-gradient methods.

Similar to what was done for reflectivity estimation, we chose $\tau_{\mathbf{Z}}$ by first generating depth images using $\tau_{\mathbf{Z}} = \beta_{\mathbf{Z}}/(1-\beta_{\mathbf{Z}})$ for each $\beta_{\mathbf{Z}} \in \{0.1, 0.2, 0.3, \ldots, 0.9\}$. and then choosing the $\tau_{\mathbf{Z}}$ value that minimized the ℓ_2 distance from the ground truth depth. As before, cross-validation methods can be employed when ground truth is unavailable.

In practical imaging scenarios, the camera will collect extremely low levels of back-reflected laser light at pixels where reflectors have very low reflectivity or are very far away. By covering the wall behind our mannequin and flower with a black cloth, we simulated such an environment in our experiments. Conventional depth estimates, even with a high number of detections per pixel, will be very noisy at such pixels because most of those pixels' detections will be extraneous. Because our Step 2 is highly effective in pixelwise censoring extraneous detections, the regularization and inpainting that we employ in our Step 3 yields depth images with sharp edges in such difficult imaging scenarios.

Note that our three-step algorithm does not exploit any correlation of reflectivity and depth. An extension of the algorithm may use the estimated reflectivity image directly for depth estimation to exploit the depth-reflectivity correlations of natural scenes [14, 15]. For example, we can define depth regularization parameters for each patch, so that higher penalization for spatial smoothness is allowed for darker regions where photon data are limited. Also, by using the final depth estimate to inform the reflectivity estimation, we are able to better correct for background-light plus dark counts. However, by exploiting depth-reflectivity correlations, we are defining a large set of parameters and iterating over the entire sequence of steps of our computational algorithm, and thus trading off computational efficiency for accuracy.

Supplementary Note 2: Photon detection-time signal-to-noise ratio

To quantify the quality of raw photon detection-time data, we will assume short-duration time bins ($\Delta/T_{\mathrm{r}} \to 0^+$), and let T be the continuous time at which a photon detection occurs at a SPAD pixel with scene object at a distance of

z meters. Its signal-to-noise ratio (SNR) in decibels (dB) is defined as

$$\mathrm{SNR} = 20\log_{10}\left(\frac{\mathbb{E}[T]}{\sqrt{\mathrm{Var}(T)}}\right), \tag{12}$$

in terms of the mean and the variance of T. The probability density function of T can then be written as

$$f_T(t;t_0) = p\tilde{s}(t-t_0) + (1-p)\mathbb{1}_{[0,T_\mathrm{r})}(t), \tag{13}$$

where $t_0 = 2z/c$, $\tilde{s}(t)$ is the normalized (unity area) photon-flux pulse waveform, p is the probability of detecting a photon from the backreflected pulse, and $\mathbb{1}_{[0,T_\mathrm{r})}(t)$ is an indicator function on the interval set $[0, T_\mathrm{r})$. Then, using the formula for computing moments of mixture densities [16], the SNR value can be computed and simplified to yield

$$\mathrm{SNR} = 20\log_{10}\left(\frac{pt_0 + (1-p)T_\mathrm{r}/2}{\sqrt{pT_\mathrm{p}^2 + (1-p)T_\mathrm{r}^2/12 + p(1-p)(t_0 - T_\mathrm{r}/2)^2}}\right). \tag{14}$$

Assume $p = 0.5$ as in our experimental setup. For the case when the object is placed at $cT_\mathrm{r}/4$, which is the middle of our depth interval $[0, cT_\mathrm{r}/2)$, we have $\mathrm{SNR} = 7.76\,\mathrm{dB}$. However, when the object is close to the sensor ($t_0 \to 0^+$), we see that the SNR of the single-photon detection can be as poor as $-2.23\,\mathrm{dB}$.

Supplementary Methods

SPAD detector array

The 32×32-pixel single-photon avalanche photodiode (SPAD) array was developed by F. Villa *et al*. at the Politecnico di Milano [17, 18, 19]. It has two modes of operation: counting mode and timing mode. In counting mode, a 6-bit counter located at each detector site is incremented each time a photon is detected, allowing for up to 63 photon detections before rolling over to 0. In timing mode, the SPAD camera outputs a digital signal at the start of each acquisition window for synchronization with external devices and for time stamping photon detection events. An internal clock running at 160 MHz controls the timing signals of the SPAD array, and each pixel has a time-to-digital converter (TDC) comprising 10 bits of timing resolution at 390 ps [18]. The time jitter of our SPAD array is about 280 ps RMS (i.e., 660 ps FWHM). In our experiment, we operated the SPAD array in the timing mode for simultaneous depth and reflectivity imaging.

Data-acquisition timing terminology and rationale

Supplementary Figure 3 illustrates the terminology and time scales for the timing-mode acquisition process. Acquisition begins when the camera issues the trigger signal to the laser diode—for synchronizing its pulse generation schedule—and ends 400 ns later, i.e., the acquisition window set by the TDC's 10-bit timing resolution combined with its $\Delta = 390$ ps time-bin duration. The TDC timing results at each pixel are stored locally in a 10-bit memory, and the data from all 1024 memory registers are then transferred to an off-chip random access memory (RAM) from which they are routed to a computer for processing. During the 10 μs needed for this data transfer, no photon detections can be recorded. The resulting 4% duty cycle for a single acquisition-readout cycle severely impacts measurements that require quick acquisition of a large amount of data. In particular, our laser's $T_\mathrm{r} = 50$ ns pulse repetition period implies that only 8 laser pulses are sent in an acquisition window, making our experiment's effective pulse rate 800 kHz, instead of 20 MHz, if each acquisition window were immediately followed by data readout.

The camera has a work-around provision, called readout-skip (RS) mode, that allows acquisition windows to be strung together without gaps by holding off the long readout process. In the experiment we adjoin 40 windows to form a

single continuous gate-on period of 16 μs. The gate-on period is followed by a 49 μs gate-off period, for limiting chip power dissipation and for reading out and transferring data from 1024 local memory registers to the computer. As indicated in Supplementary Figure 3, a full cycle of a gate-on period and the subsequent gate-off period constitutes a 65 μs frame. In our experiment we acquired 1.06 s of imaging data—comprised of 16,384 frames—for each camera position in a high-precision scan. These scans, which were performed with a two-axis translation stage, formed a 384×384 pixel composite image, as detailed in the next section.

There is a compromise inherent in operating the SPAD camera in its RS mode: suspending readout until the end of the gate-on period means that at most one photon detection per frame can be recorded at any given pixel, and this limitation could have consequences for depth and reflectivity imaging. Consider a photon detection recorded at a particular pixel in a particular frame. RS-mode operation precludes our knowing in which of that frame's 40 acquisition windows the detection event occurred, even though the 10-bit timing information within that window is intact. Because the laser's pulse-repetition period is chosen to satisfy $cT_{\mathrm{r}}/2 > z_{\max}$, where $z_{\max}$ is the scene's maximum depth, each detection of a back-reflected photon is due to the immediately preceding laser pulse. Hence the quality of timing data collected with RS-mode enabled is the same as would be obtained—over a much longer image-acquisition time—with that mode disabled.

The situation is different for reflectivity measurements, because the number of preceding laser pulses before the detection event within a frame yields useful reflectivity information. It follows that many frames are needed to acquire the first detection event. RS-mode operation records the frame in which each detection event occurs, but neither the window within that frame in which it occurred, nor the laser pulse within that window which produced that detection. In other words, we retain coarse timing information (number of frames before the detection event happens) but lose the finer timing details (number of laser pulses preceding the detection event within the frame). We operate in a low-flux scenario, however, so the probability of obtaining a detection event at a particular pixel within a frame of 40 windows is very low. The low-flux condition ensures that the probability of detection in only one window is much larger than the probability of detections from multiple windows within the same frame, and thus RS-mode operation has minimal adverse impact on reflectivity estimation in our experiments.

Imaging setup and spatial scanning

A diagram of the experimental setup is shown in Supplementary Figure 1. The

SPAD array occupies a 4.8×4.8 mm footprint that contains 32×32 pixels. We would like to have a larger imaging area, as well as more pixels, in order to more fully explore computational reconstruction methods. Given the limited size of the current SPAD array, we opted to stitch together multiple image acquisitions to form a larger-size composite image. To do so we mounted the SPAD array on a feedback-controlled, two-axis motorized translation stage and translated the array in a square pattern of 6×6 seamless tiles, as shown in Supplementary Figure 2(a), giving us a total imaging area of 28.8×28.8 mm and a resolution of 192×192 pixels. The 30 μm diameter of each pixel's circular active area being less than half the 150-μm pixel pitch provided a way to increase resolution with the same image area by translating the SPAD array along its horizontal (x) and vertical (y) axes to all combinations of 0 and 1/2 pixel-pitch distances before moving on to the next tile, as shown in Supplementary Figure 2(c). In other words, we combined 6×6 tiling with factor-of-2 sub-pixel sampling by collecting data at $(x, y) = (L_x + \Delta_x, L_y + \Delta_y)$ for all combinations of $L_x, L_y \in \{0, 4.8, 9.6, 14.4, 19.2, 24\}$ and $\Delta_x, \Delta_y \in \{0, 0.075\}$, where all dimensions are in mm. This gave us a total imaging resolution of $32 \times 6 \times 2 = 384$ pixels along each axis and required a total of $6 \times 6 \times 2 \times 2$ = 144 image acquisition sequences to produce the resulting 384×384 pixel image. Although this approach required scanning, it was still significantly faster than raster scanning with a single-pixel SPAD, which would have required a total of $384^2 = 147,456$ image acquisition sequences for the same resolution.

To obtain sufficient data for comparison with conventional imaging methods, we configured the SPAD camera to output a total of 16,384 frames for each acquisition sequence, which at 65 μs per frame took 1.06 s. For our main low-light imaging results of the mannequin and sunflower in the manuscript, we used only 20 frames, which translates to 1300 μs (320 μs gate-on reading time and 980 μs gate-off cooling time) worth of photon count data. MATLAB control programming was used to automate the acquisition procedure and synchronously drive the translation stage to each of the 144 camera positions, outputting a separate file for each position containing photon detection data. For efficient data parsing, we developed a custom GNU C program to read the SPAD array binary files and output a file containing only a sequence of photon detections tagged by pixel number and time location number. The C program also has both ASCII and binary output modes, which are conveniently and efficiently read in MATLAB. The desktop computer used for data acquisition and parsing had an Intel(R) Core(TM) i7-3840QM CPU running at 2.80 GHz.

The entire experimental data collection took tens of minutes. This long col-

lection time was due to (1) the two-axis mechanical translation, which increases the image size from 32 × 32 to 384 × 384 pixels, for increased imaging resolution, and (2) our desire for a reference image with ∼550 photon detections at every pixel for ground truth comparison and analysis. In practical applications of our methods, the reference image would not be generated and only the low-flux dataset would be used. If we did not choose to use mechanical translation to increase the imaging resolution, it would be possible to acquire a 32 × 32 image with an average of one signal photon detection per pixel using an acquisition time of $(65\ \mu\text{s per frame}) \times (20\ \text{frames}) = 1300\,\mu\text{s}$. This allows our SPAD-camera imaging system to acquire data at a frame rate of at least $769\,\text{Hz}$, which is above the video rate, but the per-frame image resolution would be limited to 1024 pixels when using our current SPAD camera. For video-rate single-photon imaging at higher resolutions, our computational framework can be applied to datasets collected by new single-photon sensors that have $\sim 100,000$ pixels [20].

Mounted in front of the translatable SPAD array was a standard Canon FL-series photographic lens with a focal length of 55 mm and an $f/1.2$ maximum photographic aperture. We set the lens' aperture to $f/2.8$, which provided depth of field sufficient to capture details from the object depths in our scene, increased sharpness, and reduced vignetting. The lens, designed for 35 mm film cameras, had an image-circle diameter slightly larger than 35 mm, allowing us to mount the lens at a fixed position and conveniently fit our $24 \times 24\,\text{mm}$ imaging area entirely within the lens' image circle. Preliminary tests in counting mode showed that at $f/2.8$ the lens was able to easily and clearly resolve objects as small as one pixel in the 384×384 pixel image with almost zero cross-talk.

Supplementary References

[1] McCarthy, A. *et al.* Long-range time-of-flight scanning sensor based on high-speed time-correlated single-photon counting. *Appl. Optics* **48**, 6241–6251 (2009).

[2] Shin, D., Kirmani, A., Goyal, V. K. & and Shapiro, J. H. Computational 3D and reflectivity imaging with high photon efficiency. In *IEEE International Conference on Image Processing (ICIP)*, 46–50 (2014).

[3] Shin, D., Kirmani, A., Goyal, V. K. & Shapiro, J. H. Photon-efficient computational 3D and reflectivity imaging with single-photon detectors. *IEEE Trans. Comput. Imaging* **1**, 112–125 (2015).

[4] Rudin, L. I., Osher, S. & Fatemi, E. Nonlinear total variation based noise removal algorithms. *Physica D: Nonlinear Phenomena* **60**, 259–268 (1992).

[5] Shen, J. & Chan, T. F. Mathematical models for local nontexture inpaintings. *SIAM J. Appl. Math.* **62**, 1019–1043 (2001).

[6] Harmany, Z. T., Marcia, R. F. & Willett, R. M. This is SPIRAL-TAP: Sparse Poisson intensity reconstruction algorithms—theory and practice. *IEEE Trans. Image Process.* **21**, 1084–1096 (2012).

[7] Tibshirani, R. Regression shrinkage and selection via the lasso. *J. Royal Statist.Soc., Series B (Methodological)* **58**, 267–288 (1996).

[8] Beck, A. & Teboulle, M. A fast iterative shrinkage-thresholding algorithm for linear inverse problems. *SIAM J. Imag. Sci.* **2**, 183–202 (2009).

[9] Golub, G. H., Heath, M. & Wahba, G. Generalized cross-validation as a method for choosing a good ridge parameter. *Technometrics* **21**, 215–223 (1979).

[10] Acton S. T. & Bovik, A. C. Piecewise and local image models for regularized image restoration using cross-validation. *IEEE Trans. Image Process.* **8**, 652–665 (1999).

[11] Liao, H. & Ng, M. K. Blind deconvolution using generalized cross-validation approach to regularization parameter estimation. *IEEE Trans. Image Process.* **20**, 670–680 (2011).

[12] Pati, Y. C., Rezaiifar, R. & Krishnaprasad, P. S. Orthogonal matching pursuit: Recursive function approximation with applications to wavelet decomposition. In *Conf. Rec. 27th Asilomar Conf. Sig., Sys., & Comput.*, vol. 1, 40–44 (Pacific Grove, CA, 1993).

[13] Kirmani, A. *et al.* First-photon imaging. *Science* **343**, 58–61 (2014).

[14] Yang, Q., Yang, R., Davis, J. & Nistér, D. Spatial-depth super resolution for range images. In *IEEE Conference on Computer Vision and Pattern Recognition (CVPR)*, 1–8 (2007).

[15] Tosic, I. & Drewes, S. Learning joint intensity-depth sparse representations. *IEEE Trans. Image Process.* **23**, 2122–2132 (2014).

[16] Frühwirth-Schnatter S. *Finite Mixture and Markov Switching Models*. (Springer Science & Business Media, 2006).

[17] Villa, F. *et al.* SPAD smart pixel for time-of-flight and time-correlated single-photon counting measurements. *IEEE Photonics J.* **4**, 795–804 (2012).

[18] Villa, F. *et al.* CMOS imager with 1024 SPADs and TDCs for single-photon timing and 3-D time-of-flight. *IEEE J. Sel. Top. Quantum Electron.* **20**, 3804810 (2014).

[19] Lussana, R. *et al.* Enhanced single-photon time-of-flight 3D ranging. *Opt. Express* **23**, 24962–24973 (2015).

[20] Veerappan, C. *et al.* A 160$\times$128 single-photon image sensor with on-pixel 55ps 10b time-to-digital converter. In *IEEE International Solid-State Circuits Conference (ISSCC)*, 312–314 (2011).
